# Linfoma Cardíaco: Um Relato de Caso

**DOI:** 10.36660/abc.20240299

**Published:** 2025-03-17

**Authors:** Leticia Ferraz Pamplona, Kelly Barnabé Serpa de Oliveira, João Ancelmo dos Reis, Luis Antelo Bustamante, Januário Manoel de Souza, Flávia Fernandes Silva Zacchi, Juliana Brenande de Oliveira Brito, Salomon Soriano Ordinola Rojas

**Affiliations:** 1 Beneficência Portuguesa de São Paulo São Paulo SP Brasil Beneficência Portuguesa de São Paulo, São Paulo, SP – Brasil; 2 Fleury Group São Paulo SP Brasil Fleury Group, São Paulo, SP – Brasil

**Keywords:** Linfoma, Neoplasias Cardíacas, Coração

## Abstract

Os tumores cardíacos são raros. O linfoma cardíaco primário é definido como um linfoma não Hodgkin, que envolve apenas o coração e/ou pericárdio. É um tumor agressivo com prognóstico ruim e seus sintomas podem ser inespecíficos. O diagnóstico definitivo só pode ser obtido através de estudo histopatológico, e a quimioterapia é a principal estratégia de tratamento.

Esse caso apresenta um paciente de 71 anos, do sexo masculino, com uma forma rara de tumor cardíaco primário. Envolvendo complicações no pós operatório como edema agudo de pulmão e necessidade de marcapasso, ocorridos após ressecção do tumor com necessidade de reconstrução de veia cava e rafia do átrio direito.

O linfoma cardíaco primário é um tumor de diagnóstico complexo por ter uma apresentação sintomática vaga e não específica, porém deve sempre ser incluído como diagnóstico diferencial de massas cardíacas. Seu diagnóstico precoce pode melhorar significativamente o prognóstico e aumentar a sobrevida desses pacientes, ao permitir um encaminhamento rápido para o tratamento específico.

## Introdução

Os tumores cardíacos são raros, porém muito agressivos. Apresentam-se em sua maioria como tumores secundários a metástases de neoplasias de pulmão, esôfago, mama, linfomas, leucemia e melanoma, com incidência de 1% em estudos de autópsias.^1 , 2^ Por outro lado, os tumores cardíacos primários são ainda mais incomuns, com incidência de aproximadamente 0,04%, variando entre 1,38 e 30 a cada 100.000 pessoas. Na maioria dos casos os tumores cardíacos primários são benignos, entre os malignos, os mais comuns são os sarcomas e linfomas, estes últimos ocorrem em cerca de 1,3% entre os tumores cardíacos primários.^2^

O linfoma cardíaco primário (LCP) é definido como um linfoma não hodgkin (LNH) que envolve apenas o coração e/ou pericárdio. Este tumor tende a comprometer o miocárdio, com maior incidência nas cavidades direitas.^3^ Podem não ser identificados no início, pois a apresentação principal é de insuficiência cardíaca (IC) de difícil tratamento. É um tumor agressivo com prognóstico ruim, que ocorre geralmente em pacientes imunossuprimidos ou imunocomprometidos.^4^

Apesar da principal forma de apresentação do LCP ser com clínica de IC, a manifestação também pode ser determinada por vários fatores como localização, velocidade de crescimento, tamanho, grau de invasão e friabilidade do tumor.^5^ Então, sintomas podem incluir dor torácica, dispneia, perda de peso, fadiga, sudorese noturna, arritmias e síndrome da veia cava superior.^1 , 2^

Exames de imagem não invasivos podem ser usados para auxiliar no diagnóstico, na identificação de massas ou complicações, porém o diagnóstico definitivo só pode ser obtido através de estudo histopatológico.^5^ A quimioterapia é a principal estratégia de tratamento após confirmação histopatológica. A radioterapia e cirurgia também são opções, mas não existem estudos que mostrem uma eficácia bem estabelecida.^2^

## Caso Clínico

Paciente de 71 anos, do sexo masculino, ex-tabagista, etilista social, portador de hiperplasia benigna da próstata e história de carcinoma basocelular em face ressecado e curado, apresentou-se em serviço de urgência com queixa de otalgia pulsátil a direita que piorava ao se deitar. Na ocasião, foi realizada lavagem otológica ipsilateral com alta hospitalar após consulta. Não se obteve melhora dos sintomas. Nos dias seguintes evoluiu com edema de face, surgimento de varizes em tórax e sensação de abafamento dos sons a direita, com nova procura ao serviço de emergência.

À observação, paciente apresentava-se em bom estado geral, estável hemodinamicamente, eupneico em ar ambiente, ausculta cardíaca e pulmonar sem achados patológicos, exame abdominal sem alterações e membros inferiores sem edemas ou sinais de trombose venosa. Apenas apresentava turgência jugular bilateral e presença de múltiplas varizes bilaterais.

**Figura 1:**
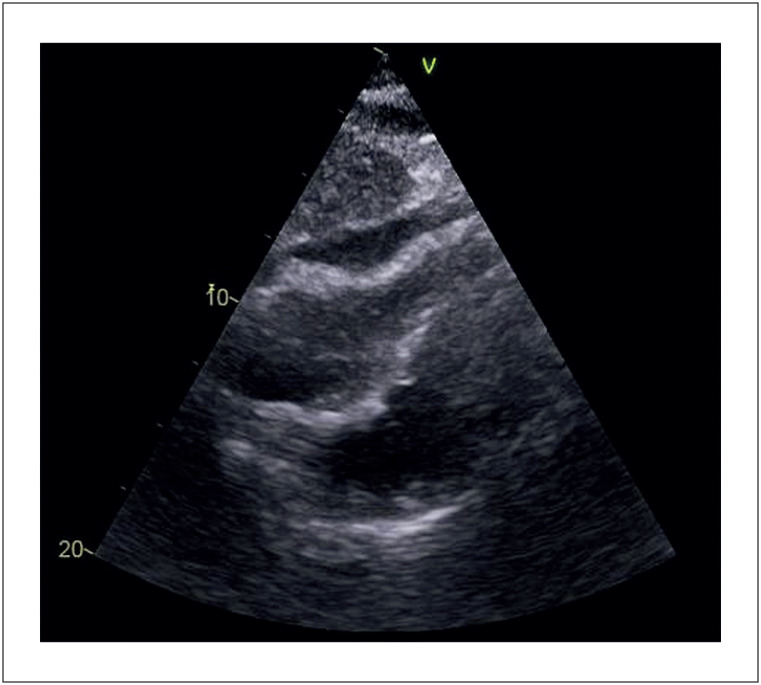
Restrição do ventrículo direito pelo derrame pericárdico observada na janela subcostal em ecocardiograma de admissão.

Foram solicitados exames laboratoriais sem alterações importantes e ecocardiograma transtorácico ( Figura 1 ) evidenciando derrame discreto e moderado envolvendo todo coração, com lâmina de 10 mm em sua maior espessura, adjacente às câmaras direitas. O exame demonstrou colapso sistólico do átrio direito além de restrição do enchimento diastólico do ventrículo direito, possivelmente decorrente ao derrame pericárdico. Fora recomendado ecocardiograma transesofágico para maior elucidação das imagens em câmaras direitas.

O paciente também foi submetido a angiotomografia de tórax e abdome, que revelou tromboembolismo pulmonar agudo, falha de enchimento no terço inferior da veia cava superior, insinuando-se discretamente pelo apêndice atrial direito (sugerindo a possibilidade de trombo hemático) e linfonodomegalias mediastinais. Nas imagens de abdome foram observados proeminência de linfonodos nas cadeias retroperitoneais, fígado exibindo imagem algo nodulariforme mal definida, hipovascular, em situação periférica no segmento 4A do lobo hepático esquerdo, além de formações hipodensas de aspecto cístico no rim direito nas imagens de abdome.

**Figura 2:**
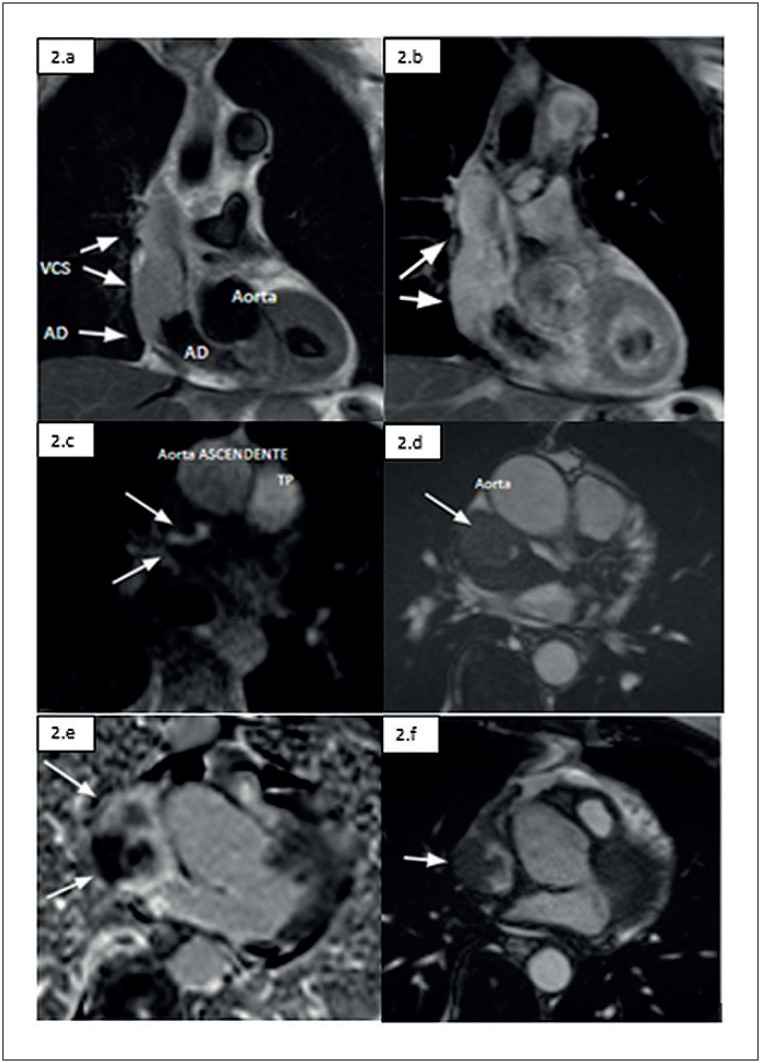
Ressonância magnética do coração evidenciando massa no interior do átrio direito (AD), ocupando praticamente todo apêndice atrial e infiltrando a porção proximal de veia cava superior (VCS): 2.a) Corte axial reto do tórax no nível da VCS: massa (setas brancas) com sinal heterogêneo na sequência *cine steady-state free precession* (cine-SSFP) 2.b) Corte axial reto do tórax no nível do apêndice atrial direito: massa (setas brancas) com sinal heterogêneo na sequência cine-SSFP. 2.c) Corte coronal do tórax seccionando o AD e a VCS: massa (setas brancas) infiltrativa com hiperssinal em T1 na sequência *Double-Invesion Recovery* (DIR). 2.d) Corte coronal do tórax seccionando o AD e VCS: massa (seta branca) infiltrativa com hiperssinal em T2 na sequência DIR 2.e) Corte axial reto do tórax no nível da VCS na sua desembocadura no AD: massa (setas brancas) infiltrativa com sinal heterogêneo na sequência de perfusão dinâmica de primeira passagem. 2.f) Corte axial reto do tórax no nível do apêndice atrial direito: massa (seta branca) com sinal heterogêneo na sequência de realce tardio.

Para maior elucidação do caso, o paciente foi encaminhado para realização de ressonância magnética cardíaca e abdominal ( Figura 2 ), com a confirmação de massa no interior do átrio direito, aderida a parede posterior e ocupando praticamente todo apêndice atrial estendendo-se até a porção proximal de veia cava superior, provocando uma obstrução significativa. E afastando a possibilidade de malignidade do nódulo hepático.

**Figura 3:**
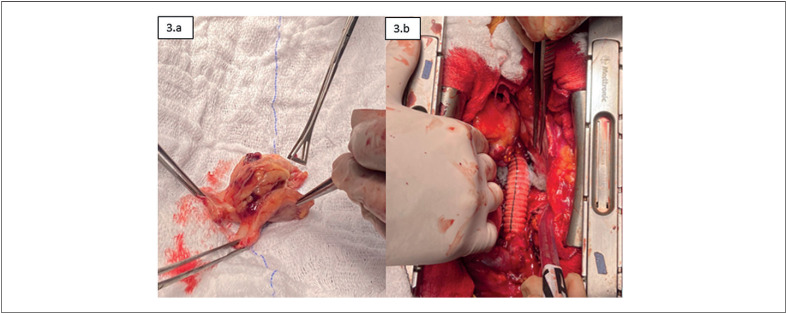
3.a) Imagem do tumor cardíaco após ressecção. 3.b) Reconstrução da veia cava superior no intraoperatório.

**Figura 4:**
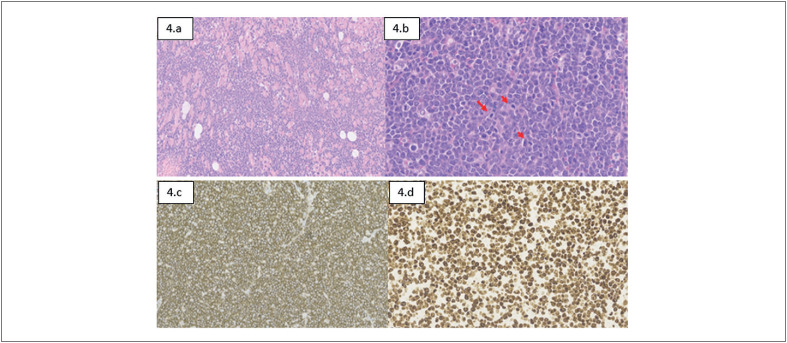
Imagens histopatológicas (Realizadas pelo sistema Leica Web Viewer), evidenciando: 4.a) Células linfoides intermediárias e grandes dissociando o interstício entre as fibras musculares cardíacas (HE – 5x) 4.b) Células linfoides intermediárias e grandes dispostas em mantos com frequentes figuras de mitose (HE – 15,5x) 4.c) Positividade forte e difusa para CD20 (CD20 – 21x). 4.d) Reação imunoistoquímica para Ki67 demonstrando alto índice proliferativo (Ki67 – 40x).

Paciente foi encaminhado para ressecção total da massa tumoral com reconstrução de veia cava superior e rafia do átrio direito ( Figura 3 ). A anatomia patológica confirmou LNH, e Imunohistoquímica apresentou achados de Linfoma Difuso de Grandes Células B subtipo Não Centro Germinativo Smile (GCB) Triplo Expressor ( Figura 4 ).

Evoluiu no pós-operatório com bradicardia juncional, sendo optado por introdução de marca-passo definitivo. Apresentou episódio de fibrilação atrial de alta resposta (FAAR) induzido por alucinações/delirium e episódio de edema agudo de pulmão (EAP) solucionados com uso de furosemida endovenosa e ventilação não invasiva (VNI). Realizado cateter venoso central de inserção periférica (PICC) e acompanhado com equipe de oncologia para início de tratamento quimioterápico.

**Figura 5:**
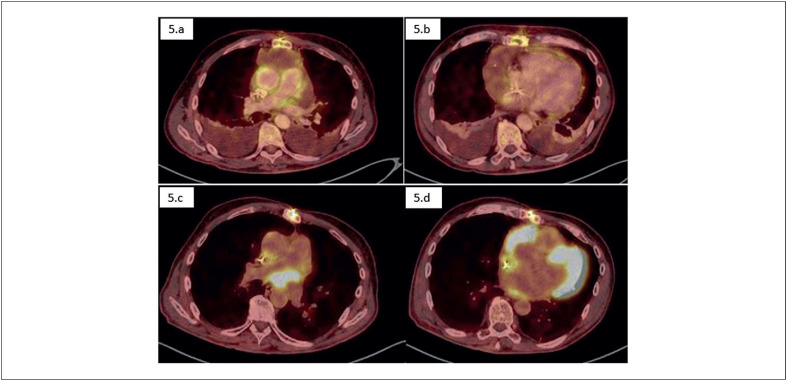
Imagens em cortes axiais de PET-SCAN usando o radiofármaco FDG que representam respectivamente: 5.a e 5.b) ausência de captações anômalas em 08/06/2023. 5.c e 5.d) captação anômala de radiofármaco evidenciando lesão expansiva hipermetabólica no leito cirúrgico atrial direito, em íntimo contato e sem planos de clivagem bem definidos com a parede lateral e posterior, além de surgimento de concentração do radiofármaco na topografia da base cardíaca em 03/01/2024.

O tratamento foi iniciado dois meses após o diagnóstico e optado pelo esquema R-CHOP (ciclofosfamida, doxorrubicina, vincristina e prednisona). No PET-CT realizado antes de início do tratamento não havia nenhuma captação anômala ( Figuras 5.a e 5.b ).

Na convalescença do 4° ciclo, o paciente apresentou grave intercorrência clínica, com hemorragia digestiva baixa/enterorragia, atribuídos à quadro de colite infecciosa, com necessidade de internação prolongada e suporte transfusional. Devido à esta grave intercorrência, decidiu-se prosseguir com os dois ciclos seguintes utilizando apenas Rituximabe, concluindo o tratamento dois meses depois.

Em PET-CT de controle, logo após finalizado o tratamento, o paciente seguia sem captações anômalas e apresentava-se completamente ativo e capaz de realizar todas as atividades realizadas anteriormente sem restrições. Porém, cerca de sete meses após, novo PET-CT mostrou nova lesão expansiva em mesmo sítio inicialmente acometido pelo Linfoma, com alta avidez pelo 18F-flúor-deoxi-2-glicose (FDG) de cerca de 6 cm, compatível com recaída da doença onco-hematológica ( Figuras 5.c e 5.d ).

O paciente optou por participar de um ensaio clínico para testar um novo quimioterápico, sendo assim, a evolução do paciente não pode ser divulgada.

## Discussão

O exame de imagem de primeira linha e mais utilizado atualmente para a avaliação de uma lesão cardíaca suspeita é o ecocardiograma transtorácico (ETT), já que fornece ótimas imagens das cavidades direitas.^3^ Existem poucos casos descritos na literatura que documentam a ação restritiva/constritiva da neoplasia, condicionando IC diastólica como mecanismo fisiopatológico subjacente.^5 - 7^ No caso em questão o Ecocardiograma Transtorácico observou colapso sistólico do átrio direito e restrição do enchimento diastólico do ventrículo direito causado pelo derrame pericárdico de 10 mm, que foi drenado posteriormente.

A ressonância magnética cardiovascular (RMC) é o método de excelência e estabeleceu-se como uma importante ferramenta na identificação e na caracterização das massas cardíacas e paracardíacas, pois é um exame não invasivo, que não utiliza radiação ionizante, é o único com a habilidade de caracterizar tecido e seu meio de contraste é o gadolíneo, mais seguro quando comparado ao contraste iodado da tomografia computadorizada.^8^ Ele permite a diferenciação dos demais diagnósticos e ainda a avaliação morfológica e funcional cardíaca em um único exame, porém exige estabilidade hemodinâmica para ser realizado.^5 , 9^ Dentre os diagnósticos diferenciais de neoplasia, destaca-se o trombo intracardíaco.^9^ No caso em questão, a RMC facilitou o diagnóstico ao observar heterogeneidade da massa no realce tardio e observar massa infiltrativa e hiperssinal em T1, T2 e na sequência de perfusão dinâmica.

O linfoma difuso de grandes células B (LDGC) é o mais comum dos Linfomas não Hodgkin (31%), sendo rapidamente fatal quando não tratado.^1^ O tratamento tem resultados melhores comparado aos demais tumores cardíacos malignos, apresentando avanços como a adição de Rituximabe (anticorpo monoclonal anti-CD20) ao tradicional esquema CHOP, permitindo o nome R-CHOP. Essa adição associou-se à melhora na sobrevida, com taxa de remissão completa de 61%.^5^

Após o tratamento, o acompanhamento cardiológico deve ser mantido. Com atenção à possibilidade de cardiotoxicidade, que pode ocorrer de forma aguda (< 14 dias), precoce (até 1 ano) e tardia (média de 7 anos). A cardiotoxicidade pode se apresentar sob forma de arritmias, síndrome coronariana aguda, miocardite, lesão endotelial miocárdica e valvar ou disfunção sistólica do ventrículo esquerdo.

Estudos^8 , 10 - 12^ demonstraram que o uso de antraciclinas, como doxorrubicina, podem causar redução de massa e leve redução da função do VE (demonstrada precocemente pelo exame ecocardiográfico de strain do VE) e maior T1 miocárdico nativo. Pacientes com alto risco desse remodelamento ventricular podem se beneficiar do uso de betabloqueadores ou inibidores da enzima conversora de angiotensina como prevenção primária. O acompanhamento desses pacientes com RMC também pode ser benéfico para avaliação precoce de função ventricular e alteração coronariana subsequente aos quimioterápicos.^8^

Esse caso representa uma forma rara de tumor cardíaco primário: envolvimento cardíaco do LDGC. Envolvendo complicações no pós-operatório como edema agudo de pulmão e necessidade de marca-passo, ocorridos após ressecção do tumor com necessidade de reconstrução de veia cava e rafia do átrio direito.

## Conclusão

O LCP é um tumor de difícil diagnóstico por se apresentar sem sintomatologia ou com sintomas vagos e inespecíficos. Porém deve sempre ser incluído como diagnóstico diferencial de massas cardíacas, pois seu diagnóstico precoce pode melhorar prognóstico e sobrevida desses pacientes, ao serem encaminhados rapidamente para tratamento específico.
