# Supplementary material for: Linfoma Cardíaco: Um Relato de Caso
Source: Arq Bras Cardiol. 2025 Mar 17;122(2):e20240299. [Article in Portuguese] doi: 10.36660/abc.20240299 (PMC12005677; doi:10.36660/abc.20240299)
Supplement: Translation

# Translation: Cardiac Lymphoma: A Case Report

**DOI:** 10.36660/abc.20240299i

**Authors:** Leticia Ferraz Pamplona, Kelly Barnabé Serpa de Oliveira, João Ancelmo dos Reis, Luis Antelo Bustamante, Januário Manoel de Souza, Flávia Fernandes Silva Zacchi, Juliana Brenande de Oliveira Brito, Salomon Soriano Ordinola Rojas

**Affiliations:** 1 Beneficência Portuguesa de São Paulo São Paulo SP Brazil Beneficência Portuguesa de São Paulo, São Paulo, SP – Brazil; 2 Fleury Group São Paulo SP Brazil Fleury Group, São Paulo, SP – Brazil

**Keywords:** Lymphoma, Heart Neoplasms, Heart

## Abstract

Cardiac tumors are rare. Primary cardiac lymphoma is defined as non-Hodgkin lymphoma that involves only the heart and/or pericardium. It is an aggressive tumor with a poor prognosis and its symptoms may be nonspecific. A definitive diagnosis can only be obtained through histopathological study, and chemotherapy is the main treatment strategy.

This case is about a 71-year-old male patient, with a rare form of primary cardiac tumor. He had postoperative complications such as acute pulmonary edema and the need for a pacemaker, which occurred after tumor resection requiring reconstruction of the vena cava and suture of the right atrium.

Primary cardiac lymphoma is a tumor that is difficult to diagnose because it has a vague and non-specific symptomatic presentation. However, it should always be included as a differential diagnosis of cardiac masses. Early diagnosis can significantly improve the prognosis and increase the survival rate of patients by quickly referring them for specific treatment.

## Introduction

Cardiac tumors are rare but rather aggressive. Occurs mostly as secondary tumors to metastases of lung, esophageal, and breast neoplasms, lymphoma, leukemia, and melanoma. They have a 1% incidence in autopsy studies.^1 , 2^By comparison, primary cardiac tumors are even rarer, with an incidence of approximately 0.04%, ranging from 1.38 to 30 per 100,000 people. In most cases, primary cardiac tumors are benign. Among malignant tumors, the most common are sarcomas and lymphomas, which occur in approximately 1.3% of primary cardiac tumors.^2^

Primary cardiac lymphoma (PCL) is defined as a non-hodgkin lymphoma (NHL) that involves only the heart and/or pericardium. This tumor tends to affect the myocardium, with a greater incidence in the right cavities.^3^It may not be identified at the beginning, as the main presentation is heart failure (HF) which is difficult to treat. It is an aggressive tumor with a poor prognosis, usually affecting immunosuppressed or immunocompromised patients.^4^

Although PCL mostly involves clinical signs of HF, its manifestation can also be determined by several factors such as location, growth rate, size, degree of invasion, and friability of the tumor.^5^Therefore, symptoms may include chest pain, dyspnea, weight loss, fatigue, night sweats, arrhythmias, and superior vena cava syndrome.^1 , 2^

Non-invasive imaging tests can be used to aid in diagnosis, by identifying masses or complications, but a definitive diagnosis can only be obtained through histopathological study.^5^Chemotherapy is the main treatment strategy after histopathological confirmation. Radiotherapy and surgery are also valid options, but there are no studies that show well-established efficacy.^2^

## Clinical case

A 71-year-old male patient, a former smoker and social drinker, with benign prostatic hyperplasia and a history of resected and healed basal cell carcinoma on the face, sought the emergency department complaining of pulsating right ear pain that worsened when lying down. At the time, ipsilateral otological lavage was performed and the patient was discharged from the hospital after a medical consultation. The symptoms did not improve. In the following days, he developed facial edema, the appearance of varicose veins in the chest, and a sensation of muffled sounds on the right, with a new visit to the emergency department.

Upon observation, the patient was in good general condition, hemodynamically stable, and eupneic in room air. His cardiac and pulmonary auscultation showed no pathological findings. Abdominal examination showed no alterations and lower limbs showed no edema or signs of venous thrombosis. He only had bilateral jugular distension and the presence of multiple bilateral varices.

Laboratory tests were requested without significant alterations and a transthoracic echocardiogram ( Figure 1 ) showed discrete and moderate effusion involving the entire heart with a 10 mm layer in its largest point (adjacent to the right chambers), in addition to systolic collapse of the right atrium and generating some restriction of diastolic filling of the right ventricle, possibly due to pericardial effusion. Transesophageal echocardiography was recommended for further elucidation of the images in the right chambers.

**Figure 1:** Right ventricular restriction due to pericardial effusion observed in the subcostal window on the echocardiogram performed upon admission.

The patient also underwent angiotomography of the chest and abdomen, which revealed acute pulmonary thromboembolism, a filling defect in the lower third of the superior vena cava, discreetly involving the right atrial appendage (suggesting the possibility of a hematic thrombus), and mediastinal lymph node enlargement. In the abdominal images, prominence of lymph nodes in the retroperitoneal chains was observed, the liver exhibiting a somewhat nodular, poorly defined, hypovascular image, in a peripheral situation in segment 4A of the left hepatic lobe, in addition to hypodense formations with a cystic appearance in the right kidney.

To further elucidate the case, the patient was referred for cardiac and abdominal magnetic resonance imaging ( Figure 2 ), which confirmed a mass inside the right atrium, attached to its posterior wall, occupying practically the entire atrial appendage and extending to the proximal portion of the superior vena cava, significantly obstructing it. This ruled out the possibility of liver nodule malignancy.

**Figure 2:** Magnetic resonance imaging of the heart showing a mass inside the right atrium (RA), occupying virtually the entire atrial appendage and infiltrating the proximal portion of the superior vena cava (SVC): 2.a) Straight axial section of the chest at the level of the SVC: mass (white arrows) with heterogeneous signal in the cine steady-state free precession (cine-SSFP) sequence. 2.b) Straight axial section of the thorax at the level of the right atrial appendage: mass (white arrows) with heterogeneous signal in the cine-SSFP sequence. 2.c) Coronal section of the chest sectioning the RA and SVC: infiltrative mass (white arrows) with hyperintensity on T1 in the Double-Inversion Recovery (DIR) sequence. 2.d) Coronal section of the chest sectioning the RA and SVC: infiltrative mass (white arrow) with hypersignal on T2 in the DIR sequence 2.e) Straight axial section of the chest at the level of the SVC at its mouth in the RA: infiltrative mass (white arrows) with heterogeneous signal in the first-pass dynamic perfusion sequence. 2.f) Straight axial section of the chest at the level of the right atrial appendage: mass (white arrow) with heterogeneous signal in the delayed enhancement sequence.

The patient was referred for total resection of the tumor mass with reconstruction of the superior vena cava and suture of the right atrium ( Figure 3 ). Pathological anatomy confirmed NHL, and Immunohistochemistry showed findings of Diffuse Large B-Cell Lymphoma, Non-Germinal Center SMILE (GCB) Triple Expressor subtype ( Figure 4 ).

**Figure 3:** 3.a) Image of the cardiac tumor after resection. 3.b) Intraoperative reconstruction of the superior vena cava.

**Figure 4:** Histopathological images (Taken by the Leica Web Viewer system), showing: 4.a) Intermediate and large lymphoid cells dissociating the interstitium between cardiac muscle fibers (HE – 5x) 4.b) Intermediate and large lymphoid cells arranged in mantles with frequent mitotic figures (HE – 15.5x) 4.c) Strong and diffuse positivity for CD20 (CD20 – 21x). 4.d) Immunohistochemical reaction for Ki67 demonstrating high proliferative index (Ki67 – 40x).

The patient developed junctional bradycardia in the postoperative period, and a decision was made to insert a permanent pacemaker. The patient had an episode of high-response atrial fibrillation (HRAF) induced by hallucinations/delirium and an episode of acute pulmonary edema (APE) resolved with the use of intravenous furosemide and noninvasive ventilation (NIV). A peripherally inserted central catheter (PICC) was placed and monitored by the oncology team to begin chemotherapy treatment.

Treatment was initiated two months after diagnosis and the R-CHOP regimen (cyclophosphamide, doxorubicin, vincristine, and prednisone) was chosen. No anomalous uptake was observed in the PET-CT scan performed before the start of treatment ( Figures 5.a and 5.b ).

**Figure 5:** Axial PET-SCAN images using the radiopharmaceutical FDG that represent respectively: 5. a and 5.b) absence of anomalous uptake on June 8, 2023. 5.c and 5.d) anomalous uptake of the radiopharmaceutical showing a hypermetabolic expansive lesion in the right atrial surgical bed, in close contact with its lateral and posterior wall and without well-defined cleavage planes. Also, there was a radiopharmaceutical concentration in the topography of the cardiac base on January 3, 2024.

During the recovery period after cycle 4, the patient had a serious clinical complication, with lower digestive hemorrhage/enterorrhagia, attributed to infectious colitis, requiring prolonged hospitalization and transfusion support. Due to this serious complication, the next 2 cycles included rituximab only, and the treatment ended 2 months later.

In a control PET-CT scan, immediately after the end of treatment, the patient continued to have no abnormal uptake and was completely active and able to perform all the activities previously performed without restrictions. However, about seven months, a new PET-CT showed a new expansive lesion in the same site initially affected by the Lymphoma, with high avidity for^18^F-fluoride-deoxy-2-glucose (FDG) of approximately 6 cm, compatible with a relapse of the onco-hematological disease ( Figures 5.c and 5.d ).

The patient chose to take part in a clinical trial to test a new chemotherapy drug and his progress cannot be disclosed.

## Discussion

The first-line and most widely used imaging test currently used to evaluate a suspected cardiac lesion is the transthoracic echocardiogram (TTE), as it provides excellent images of the right cavities.^3^There are few cases described in the literature that document the restrictive/constrictive action of the neoplasm, causing diastolic HF as the underlying pathophysiological mechanism.^5 - 7^In the case in question, the transthoracic echocardiogram showed systolic collapse of the right atrium and restriction of diastolic filling of the right ventricle caused by the 10 mm pericardial effusion, which was subsequently drained.

Cardiovascular magnetic resonance imaging (CMRI) is a method of excellence and an important tool in the identification and characterization of cardiac and paracardiac masses, as it is a non-invasive examination that does not use ionizing radiation. CMRI is the only test that can characterize tissue and its contrast medium is gadolinium, which is safer compared to the iodinated contrast used in computed tomography.^8^It allows differentiation from other diagnoses and a morphological and functional evaluation of the heart in a single examination but requires hemodynamic stability to be performed.^5 , 9^Among the differential diagnoses of neoplasia, intracardiac thrombus stands out.^9^In the case in question, CMRI facilitated the diagnosis by showing heterogeneity of the mass in late enhancement and an infiltrative and hyperintense signal mass in T1, T2, and the dynamic perfusion sequence.

Diffuse large B-cell lymphoma (DLBCL) is the most common NHL (31%), and is rapidly fatal if left untreated.^1^Treatment has better results compared to other malignant cardiac tumors, with advances such as the addition of rituximab (anti-CD20 monoclonal antibody) to the traditional CHOP regimen, having become R-CHOP. This addition has been associated with improved survival, with a complete remission rate of 61%.^5^

After treatment, cardiological monitoring must be maintained. It must consider the possibility of cardiotoxicity, which can occur acutely (< 14 days), in an early phase (up to 1 year), or a late phase (7 years on average). Cardiotoxicity can occur in the form of arrhythmias, acute coronary syndrome, myocarditis, myocardial and valvular endothelial damage, or left ventricular systolic dysfunction.

Studies^8 , 10 - 12^have shown that the use of anthracyclines, such as doxorubicin, can cause a reduction in the LV mass, a slight reduction in the LV function (demonstrated early by LV strain echocardiography), and higher native myocardial T1. Patients at high risk of this ventricular remodeling may benefit from the use of beta-blockers or angiotensin-converting enzyme inhibitors as primary prevention. Monitoring these patients with CMRI may also be beneficial for the early assessment of ventricular function and coronary changes after chemotherapy.^8^

This case represents a rare form of primary cardiac tumor: cardiac involvement induced by DLBCL. He had postoperative complications such as acute pulmonary edema and the need for a pacemaker, which occurred after tumor resection requiring reconstruction of the vena cava and suture of the right atrium.

## Conclusion

PCL is a tumor that is difficult to diagnose because it has no symptoms or has vague and non-specific symptoms. However, it should always be included as a differential diagnosis of cardiac masses, as early diagnosis can improve the prognosis and survival of patients by quickly referring them for specific treatment.
